# Preserved eHSP70-to-iHSP70 Ratio Masks a Functional Role of HSP70 in Vascular Hypercontractility in Female Diabetic Mice

**DOI:** 10.17912/micropub.biology.002146

**Published:** 2026-10-26

**Authors:** Valentina Ochoa Mendoza, Swasti Rastogi, Chloe Biby, Jessica Liaw, Kenia Pedrosa Nunes

**Affiliations:** 1 Biomedical Engineering and Science, Laboratory of Vascular Biology, Florida Institute of Technology, Melbourne, FL, US

## Abstract

Heat Shock Protein 70 (HSP70) has emerged as a modulator of vascular behavior, and its physiological role is sex- and age-dependent. In diabetic conditions, pharmacological blockade of HSP70 improves the vasculature in males. Yet, in diabetic females, the HSP70 profile and its role in vascular hypercontractility, a hallmark of type 2 diabetes (T2D), remain unknown. We showed that inhibiting aortic HSP70 in T2D females ameliorates vascular dysfunction, while the eHSP70-to-iHSP70 ratio is preserved, suggesting sex-specific differences during T2D.

**Figure 1. Female db/db aorta shows hypercontractility to Phenylephrine (PE) reversed by HSP70 inhibition, which impacts the tonic phase of contraction mediated by Ca 2+ influx, while the HSP70 ratio is preserved:** (A) Cumulative PE concentration-response curves in db/+ vehicle (grey filled circles), db/+ VER (lilac filled circles), db/db vehicle (grey unfilled circles), and db/db VER (lilac unfilled circles) aortas. (B) AUC quantification. (C) Maximal contractile response (Emax). (D) Representative time–force traces in response to a single PE challenge in db/+ (continuous lines) and db/db (dashed lines) vessels treated with vehicle (grey) or VER (lilac). (E) Emax of the phasic phase and (F) tonic phase in db/+ (filled symbols) and db/db (unfilled symbols) mice. Data in A–F are mean ± SEM; two-way ANOVA with Holm–Sidak’s post hoc test; *p ≤ 0.05, ****p ≤ 0.0001; n = 6–7. Representative immunoblot of (G) aortic iHSP70 normalized to β-actin and (H) serum eHSP70 levels normalized to total protein. (I) iHSP70/β-actin (a.u.) and (J) eHSP70/Total protein (a.u.). (K) eHSP70-to-iHSP70 ratio and (L) H index. Data in I–L are mean ± SEM; n = 4–5 mice per group in db/+ (filled circles) and db/db (unfilled circles). *p ≤ 0.05, ***p ≤ 0.001 vs. db/+, unpaired Student’s t-test or Mann–Whitney U-test as appropriate.

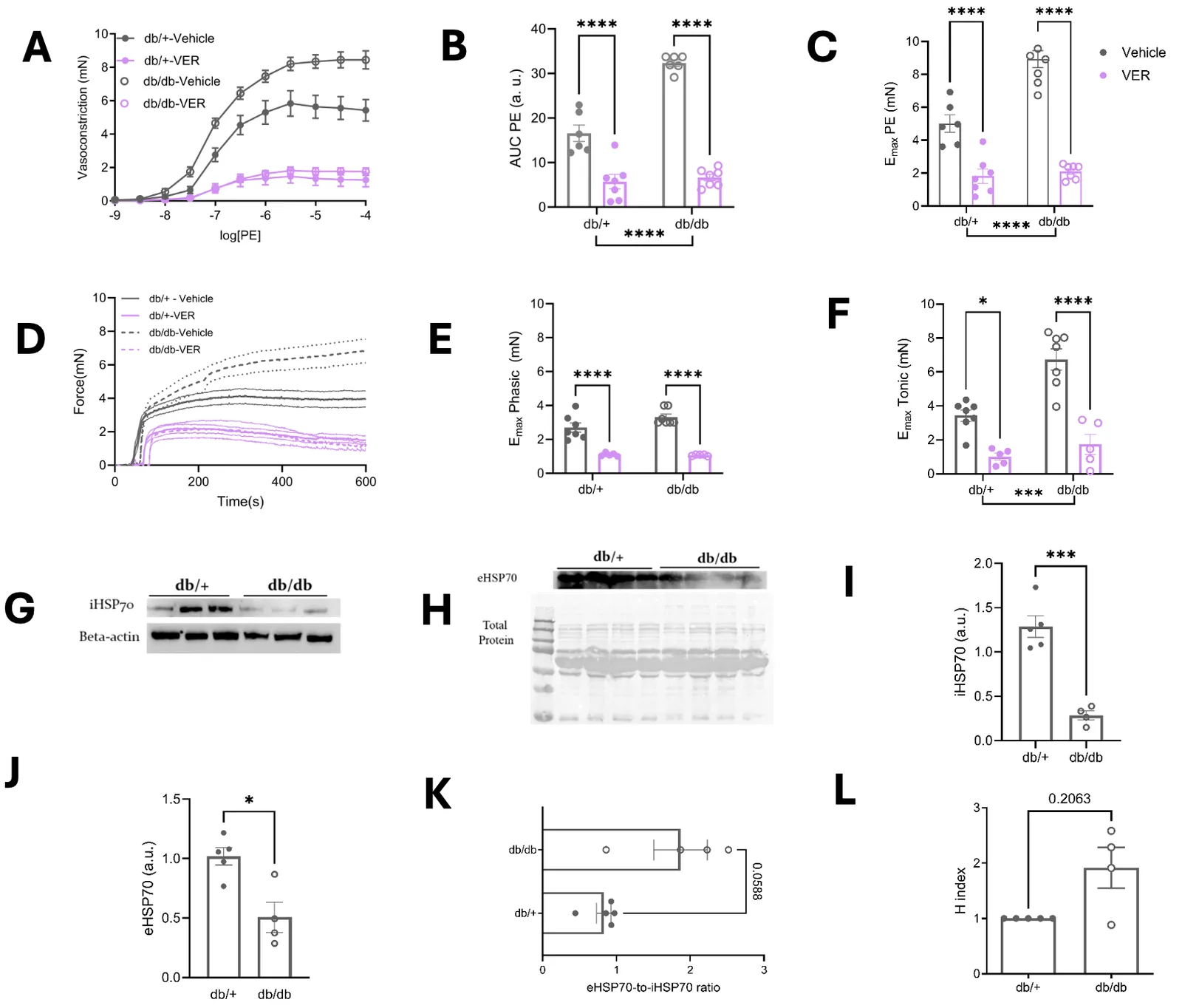



## Description

T2D-associated vascular dysfunction arises from a network of interacting mechanisms rather than a single cause, making it difficult to explain its progression through a single pathway (Dunn and Nelson, 2010; Fernández-Velasco et al., 2014). HSP70 plays critical roles across different tissues (de Oliveira et al., 2022). It is increasingly recognized as a multifunctional protein with tissue-specific roles, including important effects in muscle biology. In skeletal and cardiac muscle, loss of inducible HSP70 disrupts Ca ^2+^ homeostasis and weakens contractile performance (Kim et al., 2006; Senf et al., 2013), while in vascular smooth muscle, HSP70 supports receptor-driven contraction (de Oliveira and Nunes, 2020). Importantly, hyperglycemia has been associated with elevated circulating HSP70 levels (Nakhjavani et al., 2010), and HSP70 is now suggested to be not merely a bystander but a potential mechanistic player in this setting. HSP70 is ubiquitously expressed, residing intracellularly (iHSP70) or released extracellularly in the circulation (eHSP70), and its effects in diabetes depend critically on both location and ratio (de Oliveira et al., 2022). In this manuscript, HSP70 refers to the entire 13-isoform family, as our experimental design targets total HSP70 levels without distinguishing among variants. An imbalance between eHSP70 and iHSP70 has been proposed as an inflammatory biomarker, and a disruption of this ratio is associated with vascular dysfunction in both type 1 (de Oliveira et al., 2022) and T2D (Ochoa Mendoza et al., 2025). In the aorta of male rodents, HSP70 contributes to receptor-mediated vasoconstriction by regulating Ca ^2+^ -handling mechanisms, supporting an important role for HSP70 in maintaining physiological vascular tone (de Oliveira et al., 2021).

Recent evidence indicates that vascular HSP70 expression and function are age- (de Oliveira et al., 2022) and sex-dependent (de Oliveira et al., 2021). Healthy female rats have lower aortic HSP70 levels than males, despite reports of higher HSP70 expression in some female tissues such as the heart (Voss et al., 2003). Under non-pathological conditions, pharmacological blockade of HSP70 with VER155008 has a greater impact on phenylephrine-induced contraction in female aortic rings than in male rings, affecting both phasic and tonic components of the response (de Oliveira et al., 2021). VER155008 is a competitive HSP70 inhibitor that binds to the ATPase pocket of HSP70, preventing ATP hydrolysis (Williamson et al., 2009), without compromising vessel viability (De Oliveira and Nunes, 2020). These findings suggest that impaired vascular HSP70 expression in females increases their dependence on HSP70-mediated Ca²⁺ handling to maintain contractile tone, providing a potential molecular axis for sex-specific differences in vascular reactivity.

Given that females are generally protected from cardiovascular disease for much of their lives but may lose this advantage in T2D, and HSP70 might be a possible therapeutic target (de Oliveira et al., 2019), we examined the contribution of HSP70 to vascular dysfunction in a T2D diabetic female (db/db) model. Our findings show that (1) the hypercontractile phenotype in the aorta of T2D females is prevented by HSP70 inhibitor VER155008, similar to results in males, (2) but it only impacts the tonic phase of contraction when comparing control (db/+) versus diabetic (db/db), and (3) the eHSP70-to-iHSP70 ratio is preserved in T2D females, contrasting with data in males (Ochoa Mendoza et al., 2025).

In the aorta of female db/db mice, pharmacological inhibition of HSP70 showed a more pronounced effect than in control db/+ mice ( Figure 1A- C), indicating that HSP70 contributes to the exaggerated vasoconstriction observed in T2D. Considering that HSP70 blockade reduces Ca²⁺ influx–mediated contraction and lowers free cytosolic Ca²⁺ levels in aortic tissues from T2D males (Ochoa Mendoza et al., 2025), we evaluated the biphasic contraction curve in response to a single dose of PE in the aorta of diabetic females. This biphasic behavior reflects a two‑phase rise in cytosolic Ca²⁺. The phasic phase is driven predominantly by IP₃ receptor (IP₃R) ‑mediated Ca²⁺ release from the SR, whereas the tonic phase depends on continued Ca²⁺ influx through voltage‑dependent and receptor‑operated channels, together with Ca²⁺‑sensitizing mechanisms that maintain force at relatively sustained Ca²⁺ levels (Webb, 2003). HSP70 inhibition reduced contraction in both phases of the biphasic curve ( Figure 1D, 1E, and 1F) in db/+ and db/db vessels. However, a significant difference between the db/+ and db/db groups was observed only during the tonic phase ( Figure 1F ), suggesting impaired Ca²⁺ influx. The aorta's vascular reactivity corroborates the hypercontractile profile triggered by diabetes, which HSP70 inhibition reduces, thereby improving overall vascular function in T2D females.

Circulating eHSP70 is suggested as a target for the treatment of diabetic vasculopathies, as it can act like a damage-associated molecular pattern (DAMP) and lead to an innate immune response via TLR4 (de Oliveira et al., 2022), thus it is associated with low-grade inflammation, whereas increased iHSP70 is regarded as protective and anti-inflammatory (de Oliveira, et al., 2022). A disruption in the balance between eHSP70 and iHSP70 can lead to vascular dysfunction in diabetic males (Ochoa Mendoza et al., 2025). Still, the HSP70 ratio was unknown in adult females. Therefore, we evaluated aortic and systemic HSP70 levels. Importantly, a limitation here is that HSP70’s tissue levels include both cytosolic and membrane-bound HSP70. Our data showed that both iHSP70 and eHSP70 were reduced in control db/+ versus diabetic db/db ( Figure 1 G-J), which led to an undisrupted eHSP70-to-iHSP70 ratio (Krause et al., 2015) and H index (Costa-Beber et al., 2020), both used as indirect biomarkers for low-grade inflammation ( Figure 1K and L, respectively), differing from male diabetic mice previously reported (Ochoa Mendoza et al., 2025).

An important mediator of sex differences in cardiovascular function across species is the nitric oxide (NO)/NO synthase (NOS) pathway. Although the molecular mechanisms underlying these sex differences remain incompletely defined, the NO/NOS pathway has been strongly implicated in both humans (Forte et al., 1998; Sader and Celermajer, 2002) and rats (Kauser and Rubanyi, 1994). In humans, premenopausal women display superior NO-dependent endothelial vasodilation compared to men, attributable to estrogen's ability to upregulate eNOS expression, enhance its phosphorylation, and boost NO bioavailability while mitigating its oxidative inactivation (Steinberg et al., 2000; White, 2002). Rodent models corroborate this pattern, with female rats exhibiting elevated basal NO production and greater NO-mediated vascular relaxation than males (Kauser and Rubanyi, 1994).

These estrogen-driven effects not only promote endothelium-dependent relaxation but also likely influence chaperone homeostasis by curbing hyperglycemia-induced inflammation and oxidative stress, which can otherwise disrupt the eHSP70-to-iHSP70 balance according to the chaperone balance hypothesis (Krause et al., 2015). The preserved eHSP70:iHSP70 ratio and H index in our diabetic females may reflect a more stable, coordinated adaptation to diabetic stress. Notably, a disrupted ratio has been reported only in postmenopausal T2D women, corroborating that at the adult stage, our females are still protected (Seibert et al., 2022). In T2D, where metabolic stressors typically compromise NO signaling, one could say that the preserved HSP70 equilibrium in the female db/db mice suggests a lingering protective NO/NOS axis. This may explain the exaggerated attenuation of hypercontractility by VER155008 in diabetic females (Figure A and D, lilac lines), as HSP70 inhibition could synergize with residual NO-mediated vasodilation to more effectively counteract sustained vascular tone. Interestingly, under physiological conditions, HSP70 inhibition does not affect NO in the vasculature of males (de Oliveira and Nunes, 2020), which does not exclude the possibility that this landscape changes in females. Overall, the data presented corroborate HSP70’s involvement in vascular function, expand our understanding of HSP70's sex-specific role in pathological conditions, and highlight a worthwhile therapeutic window for targeting HSP70 to restore vascular function in women with T2D.

## Methods

*Ex vivo assessment of thoracic aortic function by wire myograph*

Thoracic aortic rings (2 mm in length) from db/db and db/+ female mice were mounted in a DMT620M multi-wire myograph system (Danish Myo Technology, Copenhagen, Denmark) under isometric conditions and set to an optimal resting tension of approximately 5 mN in PSS, continuously aerated with carbogen (95% O _2_ , 5% CO _2_ ) at 37 °C. The protocol for the equilibration period and verification of functional integrity of each ring was assessed as previously described (Ochoa Mendoza et al., 2025).

*Cumulative concentration-response curves*

After equilibration and viability confirmation, the aortic rings were incubated for 30 min with vehicle (DMSO) or VER155008, a small-molecule HSP70 inhibitor (10 μmol/L in DMSO; Sigma Aldrich, St. Louis, MO, USA; catalog SML0271). The cumulative concentration-response curves for phenylephrine (PE; 1 nmol/L-100 μmol/L) were then generated, with contractile responses recorded at each concentration. Area under the curve (AUC) was calculated using the trapezoidal rule.

*Time-force curves*

For time-force analysis, aortic segments were incubated for 30 min with vehicle (DMSO) or VER155008 (10 µmol/L), followed by exposure to a single dose of PE (10⁻ ^6^ mol/L). Isometric force was recorded continuously for 15 min (1 s sampling rate). Time-force curves were fit to a bi-exponential function using least-squares regression:

*F (t) = F _Fast_ ( 1 - e ^-^* ^k^ *^_Fast_ x t^ ) + F _Slow_ ( 1 - e ^-^* ^k^ *^_Slow_ x t^ )*

The phasic component was quantified as the peak force at the fast-to-slow transition (approximated as F _fast_ ), and the tonic component as the sustained force at 15 min (approximated as F _slow_ ), both expressed relative to baseline (time 0).

*Western blot analysis of HSP70*

Thoracic aortas were homogenized in Tissue Protein Extraction Reagent supplemented with protease inhibitors, and protein concentration was determined by BCA assay. Equal amounts of aortic protein (10 µg) or serum (10 µL) were separated by 10% SDS-PAGE and transferred to nitrocellulose membranes. Membranes were briefly stained with Ponceau S to confirm transfer, washed in TBS-T, and blocked for 1 h at room temperature in 5% non-fat dry milk. They were then incubated overnight at 4 °C with a HSP70 primary antibody (Cell Signaling Technology, Danvers, MA, USA, #4872), followed by 1 h at room temperature with an HRP-conjugated secondary antibody (Cell Signaling Technology, Danvers, MA, USA, #7074). Signal was developed using a chemiluminescent substrate and detected with a ChemiDoc MP imaging system. Band intensity was quantified in ImageJ and normalized to β-actin for aortic samples. Serum samples were normalized to total protein load, since housekeeping proteins are not suitable for this matrix.

*Statistical analysis*

Statistical analyses and graphing were performed with GraphPad Prism 10.3.1 (GraphPad Software, San Diego, CA, USA). Data are presented as means ± SEM. Normality was assessed using the D’Agostino–Pearson test, and outliers were identified and excluded with Grubb’s test when appropriate. Two-group comparisons were performed using unpaired Student’s t-tests or Mann–Whitney U-tests, depending on data distribution, whereas multiple-group analyses were conducted by two-way ANOVA followed by Sidak’s post hoc test. Statistical significance was set at *p ≤ 0.05. Sample size (n) indicates the number of mice per group.

## Reagents

Female db/db mice (B6.BKS(D)-Leprdb/J) and age-matched heterozygous db/+ controls were purchased from The Jackson Laboratory (Bar Harbor, ME, USA; n = 20 total). Body weight was recorded, and non-fasting blood glucose were measured biweekly with a Metene TD-4116 glucose monitor (TaiDoc Technology Corporation, New Taipei City, Taiwan). Diabetic profile was confirmed by hyperglycemia (350 mg/dl or higher). Mice were anesthetized with 4% isoflurane in 100% O _2_ , and blood was collected from the abdominal aorta into BD Vacutainer serum tubes. Serum was isolated, snap-frozen in liquid nitrogen, and later used for eHSP70 analysis by Western blotting. Animals were then euthanized at 16 weeks by exsanguination via cardiac puncture, and thoracic aortas were harvested for snap-freezing or vascular reactivity studies in the myograph. All procedures were approved by the Institutional Animal Care and Use Committee of the Florida Institute of Technology (protocol #2022-04) and performed under veterinary supervision in accordance with ARRIVE guidelines.
